# Hyperselective Neurectomy for Upper-Limb Spasticity Under General Anesthesia or Wide-Awake Surgery: A Retrospective Comparative Cohort Study

**DOI:** 10.2106/JBJS.OA.26.00299

**Published:** 2026-10-27

**Authors:** J. Terrence Jose Jerome, Ganesan Surendran, Kuppusamy Thirumagal

**Affiliations:** 1 Olympia Hospital & Research Centre, Trichy, Tamil Nadu, India; 2 Trichy SRM Medical College, Trichy, Tamil Nadu, India

## Abstract

**Background:**

Hyperselective neurectomy (HSN) reduces focal upper-limb spasticity while preserving voluntary function. Wide-awake surgery allows intraoperative assessment of tone and movement, but comparative evidence is limited.

**Methods:**

We retrospectively studied 44 patients undergoing upper-limb HSN under general anesthesia (general anesthesia [GA]; n = 23) or wide-awake local anesthesia without a tourniquet (n = 21). Allocation was nonrandom, and wide-awake surgery was limited to patients able to cooperate with intraoperative testing. Mean follow-up was 23 months after GA and 24 months after wide-awake surgery. The patient was the primary unit of inference; nonindependent joint-level outcomes were summarized descriptively.

**Results:**

All wide-awake procedures were completed without conversion to GA (0%; exact 95% confidence interval [CI], 0%–16.1%). Intraoperative assessment prompted modification of the neurectomy plan in 5 patients (23.8%; 95% CI, 8.2%–47.2%). Mean House classification improved from 2.0 (SD, 1.0) to 3.8 (SD, 0.9) after GA and from 2.1 (SD, 0.9) to 4.4 (SD, 0.8) after wide-awake surgery; final patient-level distributions differed on rank-based comparison (p = 0.02). Both cohorts showed reduced spasticity and improved motion. Final elbow, forearm, and wrist active arcs were numerically greater after wide-awake surgery, whereas thumb arcs were similar. No major complications or reoperations occurred.

**Conclusions:**

Wide-awake HSN was feasible in selected cooperative patients and enabled intraoperative modification of the surgical plan. Although some outcomes numerically favored wide-awake surgery, the small, nonrandomized cohorts, selective eligibility, temporal and learning-curve effects, adjunct procedures, and clustered observations preclude causal or superiority conclusions.

**Level of Evidence:**

Level III (retrospective comparative cohort study). See Instructions for Authors for a complete description of levels of evidence.

## Introduction

Hyperselective neurectomy (HSN) has traditionally been performed under general anesthesia (GA), with durable reductions in spasticity reported in adult and pediatric populations^1-5^. When neuromuscular blockade is minimized or avoided, intraoperative neurostimulation identifies motor branches supplying overactive muscles. More recently, wide-awake local anesthesia without a tourniquet has been used for selected spasticity procedures. Preservation of tone and voluntary movement permits incremental fascicular titration, immediate functional reassessment, and modification of the operative plan when residual imbalance is identified^6^. Existing evidence otherwise consists largely of heterogeneous adult and pediatric series performed predominantly under general anesthesia^7-17^ (Supplementary Table I, Appendix).

Direct comparisons of GA and wide-awake HSN are limited. We asked (1) what proportion of selected patients completed wide-awake HSN without conversion to GA or a major perioperative complication; (2) at approximately 2 years, what were the between-group differences in patient-level outcomes and the descriptive patterns in joint-specific motion and spasticity; and (3) how often and in what ways did awake assessment lead to documented modification of the neurectomy plan? Because treatment allocation was nonrandom, patient-level comparisons were exploratory and were not intended to establish causality or superiority; joint-specific outcomes were descriptive.

## Methods

### Study Design and Setting

This Level III retrospective comparative cohort study included consecutive patients undergoing HSN between January 2021 and December 2023. Anesthetic allocation was nonrandom. Wide-awake surgery was offered to patients able to follow intraoperative commands and tolerate prolonged awake surgery without contraindications to local anesthesia with epinephrine. GA was used according to patient preference or when wide-awake surgery was unsuitable because of age, cognitive or communication impairment, severe anxiety, behavioral intolerance, or anesthetic contraindications.

Institutional approval was obtained for retrospective analysis of prospectively collected data. Patients or legal guardians provided written informed consent for treatment and use of deidentified data. Two fellowship-trained hand surgeons experienced in upper-limb spasticity surgery performed all procedures using consistent operative principles and rehabilitation protocols. Exact prestudy HSN case volumes could not be reliably reconstructed.

Wide-awake HSN was introduced in 2022 and progressively adopted; therefore, most wide-awake procedures occurred later in the study. Although multidisciplinary assessment, operative principles, and rehabilitation remained consistent, temporal and learning-curve effects could not be excluded. Reporting followed the STROBE guidelines.

### Participants

Inclusion criteria were upper-motor-neuron–related focal upper-limb spasticity that impaired function or care; involvement of the elbow flexors, pronators, wrist or finger flexors, or thumb; Modified Ashworth Scale (MAS) grade ≥3 in at least 1 target muscle; and retained voluntary motor control. Exclusion criteria were fixed contracture requiring bony or major soft-tissue release, absent volition, severe sensory loss, concurrent botulinum toxin injection into a target muscle, or contraindication to surgery under the planned anesthetic. Adults and children were pooled because the study was not powered for age-specific analysis.

### Cohorts and Unit of Analysis

Forty-four patients met the eligibility criteria: 23 underwent HSN under GA and 21 underwent wide-awake HSN. The patient was the primary unit of inference. Multilevel procedures generated 70 joint-level observations (35 per cohort): 11 elbow, 9 forearm-rotation, 9 wrist, and 6 thumb/digit observations in each cohort. House functional classification, recurrence, reintervention, and complications were analyzed at the patient level. Because multiple observations from individual patients were nonindependent, joint-specific outcomes were summarized descriptively by anatomic level.

### Clinical Assessment

A hand surgeon, rehabilitation physician, and occupational or physiotherapist performed standardized assessments. The MAS grades resistance to passive movement on an ordinal scale from 0 to 4, including grade 1+, with lower grades indicating less resistance. Modified Tardieu assessment recorded the fast-stretch catch angle (R1), passive range during slow stretch (R2), dynamic component (R2−R1), and muscle-reaction score. Active and passive range of motion was measured with a goniometer. Medical Research Council strength was graded from 0 to 5. House functional classification ranges from 0 (no use) to 8 (complete spontaneous use). Follow-up was scheduled at approximately 6 weeks; 3, 6, and 12 months; and annually thereafter.

### Measurement Properties and Clinical Interpretation

Study-specific interobserver reliability was not assessed. Published estimates vary by population and protocol: upper-extremity MAS reliability includes an intraclass correlation coefficient (ICC) of 0.781 (95% confidence interval [CI], 0.679 to 0.853) and κ of 0.625 (95% CI, 0.350-0.801)^18^; Modified Tardieu interrater estimates range from 0.56 to 0.851^19^; and upper-extremity goniometric ICCs range from 0.26 to 0.99^20^. House classification has demonstrated an ICC of 0.92 in children with cerebral palsy, although no 95% CI was reported and applicability to this mixed HSN cohort is uncertain ^21^. These estimates were not treated as measures of precision for the present study.

No minimal clinically important difference (MCID) has been validated after upper-limb HSN in a mixed adult and pediatric population. The reported poststroke upper-extremity MAS MCID of 0.48 to 0.76 points^22^ was used only for context, not to define treatment success, because MAS grades are ordinal and joint-level observations were nonindependent.

### Surgical Technique

#### Hyperselective Neurectomy

Under magnification, motor rami were dissected to their intramuscular entry points and confirmed by neurostimulation, with preservation of sensory branches. Approximately two-thirds of the selected motor fascicles were initially resected. Resection was increased to three-quarters when stimulation-evoked contraction or tone persisted, provided voluntary activation was preserved during wide-awake surgery or an adequate motor response remained under GA. A 5–10 mm segment was excised, leaving a fascicular remnant to preserve voluntary control.

#### General Anesthesia

GA was administered with endotracheal intubation and an upper-arm tourniquet. Long-acting neuromuscular blockade was avoided after induction to permit neurostimulation. Because voluntary movement, functional posture, and velocity-dependent spasticity could not be assessed intraoperatively, neurectomy and adjunct procedures followed the preoperative plan.

### Wide-Awake Technique

Buffered lidocaine with epinephrine was infiltrated along the planned exposure, followed by 20 to 30 minutes for vasoconstriction. The maximum lidocaine dose was calculated using body weight, comorbidities, solution concentration, and institutional policy; cumulative dose and volume were documented.

After incremental fascicular resection, neurostimulation was combined with reassessment of tone, fast-stretch catch, voluntary movement, and functional posture. An adjunct tendon or soft-tissue procedure was considered only when a reproducible dynamic deformity persisted, was consistent with preoperative goals, remained within the consented scope, and could be completed below the local-anesthetic ceiling. No patient reached this ceiling, developed clinical evidence of local-anesthetic systemic toxicity, requested termination, or underwent conversion to GA.

Neurectomy-plan modification was defined as a documented intraoperative change in the targeted motor fascicles or intended extent of resection after awake reassessment. Addition or omission of an adjunct procedure was recorded separately. Both variables were extracted from contemporaneous operative notes, which were adequate for classification in all 21 wide-awake cases; modifications were not inferred from surgeon recollection.

### Postoperative Rehabilitation

Both cohorts followed a standardized program of early protected motion, progressive antagonist activation, task-specific practice, and staged strengthening, modified when adjunct tendon procedures were performed.

### Statistical Analysis

The patient was the primary unit of inference. Baseline characteristics were summarized as mean (SD), median (IQR), or number (percentage), as appropriate. Because allocation was nonrandom, baseline differences were described without assuming cohort equivalence. Patient-level continuous, ordinal, and categorical outcomes were compared using Welch independent-samples t tests, Mann-Whitney U tests, and Fisher exact tests, respectively. Categorical effects are reported as risk differences (wide-awake minus GA) with Newcombe 95% CIs; single-cohort proportions use exact Clopper-Pearson CIs. House classification was analyzed as an ordinal outcome using the Mann-Whitney U test; means and SDs are descriptive, and no CI was calculated for the mean difference.

Because some patients underwent procedures at multiple anatomic levels, the 70 joint-level observations were nonindependent. The 6 to 11 patients per cohort at each level were insufficient for reliable clustered analyses or covariate adjustment. Joint-specific baseline and final outcomes were therefore summarized descriptively without hypothesis tests, effect estimates, CIs, or P values (Supplementary Table II, Appendix). MAS, Tardieu catch-quality, MRC, and House scores were treated as ordinal, with means and SDs presented descriptively.

No multivariable model was fitted because 44 independent patients were insufficient for stable adjustment for baseline imbalance, selection factors, chronicity, surgeon, calendar period, and adjunct procedures. Secondary patient-level analyses were exploratory, without multiplicity correction. Tests were 2-sided with α = 0.05 and performed using IBM SPSS Statistics for Windows, version 29.0 (IBM Corp).

## Results

### Baseline Characteristics

The wide-awake cohort comprised 17 adults and 4 children, and the GA cohort comprised 14 adults and 9 children (Table 1). Among patients with traumatic brain injury, mean time from injury to surgery was 2.1 years (SD, 0.5) after wide-awake surgery and 3.2 years (SD, 1.0) after GA.

**Table 1:** Patient Demographics and Baseline Characteristics by Cohort

Parameter	Wide-Awake (n = 21)	GA (n = 23)
Sex, n		
Male	13	14
Female	8	9
Age at surgery		
Adults (≥18 years), n	17	14
Adults, age, mean (SD), years	39 (15)	41 (13)
Children (<18 years), n	4	9
Children, age, mean (SD), years	12 (5)	13 (4)
Etiology, n (%)		
Traumatic brain injury	12 (57)	13 (57)
Cerebrovascular accident	4 (19)	4 (17)
Cerebral palsy	2 (10)	2 (9)
Infantile hemiplegia	2 (10)	2 (9)
Other	1 (5)	2 (9)
Extent of spasticity, n (%)		
Hemiplegia	18 (86)	19 (83)
Quadriplegia	3 (14)	4 (17)
Time from event to surgery, mean (SD), years		
Traumatic brain injury	2.1 (0.5)	3.2 (1.0)
Cerebrovascular accident	5.5 (3.0)	6.0 (2.0)
Cerebral palsy	13.0 (3.0)	15.0 (4.0)
Infantile hemiplegia	14.0 (7.0)	13.0 (6.0)

Data are presented as mean (SD) or number (percentage), as appropriate. Baseline characteristics are descriptive; nonrandomized cohorts were not assumed to be equivalent. GA indicates general anesthesia.

### Feasibility and Plan Modification

All 21 wide-awake procedures were completed without conversion to GA (0%; exact 95% confidence interval [CI], 0%–16.1%). Awake reassessment prompted modification of the targeted motor fascicles or extent of resection in 5 patients (23.8%; 95% CI, 8.2%–47.2%). No neurectomy-plan modification occurred under GA; adjunct procedures were recorded separately.

### Joint-Specific Outcomes

Each cohort contributed 11 elbow, 9 forearm-rotation, 9 wrist, and 6 thumb/digit observations. Both cohorts showed reduced spasticity and increased motion (Supplementary Table II, Appendix). Final active arcs at the elbow, forearm, and wrist were numerically greater after wide-awake surgery, whereas thumb arcs were similar. These nonindependent, small-subgroup observations were not analyzed inferentially and do not establish comparative effectiveness.

### Adjunct Procedures

Adjunct procedures were performed in 9 of 21 wide-awake patients (42.9%) and 4 of 23 GA patients (17.4%; Fisher exact P = 0.099). These included extensor carpi ulnaris-to-extensor carpi radialis brevis transfer in 4 wrist cases (2 per cohort), extensor pollicis longus rerouting in 3 wide-awake thumb cases, and extensor tenolysis in 1 thumb case per cohort. Wide-awake adjunct procedures followed intraoperative functional reassessment; GA procedures followed the preoperative plan.

### Patient-Level Outcomes and Safety

Mean House classification improved from 2.0 (SD, 1.0) to 3.8 (SD, 0.9) after GA and from 2.1 (SD, 0.9) to 4.4 (SD, 0.8) after wide-awake surgery. Final distributions differed on Mann-Whitney *U* testing (P = 0.02; Table 2); means and SDs are descriptive because House classification is ordinal.

**Table 2:** Patient-Level Outcomes After Hyperselective Neurectomy

Patient-Level Outcome	GA (n = 23)	Wide-Awake (n = 21)	Effect Estimate (95% CI)	p
Functional outcome				
House functional classification, baseline	2.0 (1.0)	2.1 (0.9)	—	—
House functional classification, final follow-up	3.8 (0.9)	4.4 (0.8)	Descriptive mean difference, +0.6; 95% CI not calculated because the House classification is ordinal	0.020
Feasibility and operative plan				
Conversion to GA	Not applicable	0/21 (0%)	Wide-awake proportion, 0% (exact 95% CI, 0% to 16.1%)	—
Documented modification of neurectomy plan	0/23 (0%)	5/21 (23.8%)	Risk difference, +23.8% (95% CI, 4.4% to 45.1%)	0.019
Adjunct procedure performed	4/23 (17.4%)	9/21 (42.9%)	Risk difference, +25.5% (95% CI, −1.5% to 48.5%)	0.099
Safety and durability				
Major complication	0/23 (0%)	0/21 (0%)	Risk difference, 0.0% (95% CI, −14.3% to 15.5%)	>0.99
Transient paresthesia	1/23 (4.3%)	3/21 (14.3%)	Risk difference, +9.9% (95% CI, −9.1% to 30.6%)	0.335
Recurrent spasticity	1/23 (4.3%)	0/21 (0%)	Risk difference, −4.3% (95% CI, −21.0% to 11.5%)	>0.99
Reintervention for recurrent spasticity	1/23 (4.3%)	0/21 (0%)	Risk difference, −4.3% (95% CI, −21.0% to 11.5%)	>0.99
Repeat HSN/reoperation	0/23 (0%)	0/21 (0%)	Risk difference, 0.0% (95% CI, −14.3% to 15.5%)	>0.99
Patient acceptance				
Willingness to undergo the same anesthetic technique again	Not collected	21/21 (100%)	Wide-awake proportion, 100% (exact 95% CI, 83.9% to 100%)	—

CI = confidence interval, GA = general anesthesia, HSN = hyperselective neurectomy.

Data are presented as mean (SD) or number/total (percentage), as appropriate. Outcomes were measured once per patient. House functional classification is ordinal; its means and mean difference are descriptive, no CI was calculated for the mean difference, and the final-follow-up P value is from the Mann-Whitney *U* test. Categorical P values are from 2-sided Fisher exact tests. Risk differences are wide-awake minus GA with Newcombe 95% CIs; exact Clopper-Pearson CIs are reported for single-cohort proportions. The neurectomy-plan-modification comparison demonstrates the technical capability of awake assessment and should not be interpreted as comparative clinical effectiveness. Secondary comparisons are exploratory; no multiplicity correction was applied.

At approximately 2 years, no patient underwent repeat surgery. One GA patient developed mild recurrent spasticity managed with botulinum toxin. No major complications occurred, although the exact 95% CI upper limits were 16.1% after wide-awake surgery and 14.8% after GA. Transient paresthesias in 3 wide-awake and 1 GA patient resolved without intervention.

### Patient Acceptance

All 21 wide-awake patients reported willingness to undergo the same anesthetic technique again. Patient-experience data were not collected after GA, precluding comparison.

## Discussion

HSN under both GA and wide-awake surgery was associated with sustained reductions in spasticity, preserved voluntary strength, and improved functional use. Wide-awake surgery enabled intraoperative reassessment and modification of the surgical plan. Although some joint-specific outcomes numerically favored wide-awake surgery, these nonindependent observations were descriptive and do not establish comparative effectiveness.

The principal technical advantage of wide-awake HSN was reassessment of tone, voluntary activation, and functional posture after incremental fascicular resection. This prompted modification of the neurectomy plan in 5 of 21 patients and permitted adjunct procedures for reproducible residual dynamic imbalance, supporting the value of preserving tone and volitional control during fascicular titration^6^. However, adjunct procedures were more frequent in the wide-awake cohort and may have influenced postoperative motion and posture. Wide-awake testing therefore represents a technical capability in selected patients, not evidence that anesthetic strategy improves recovery. GA remains appropriate for patients unable or unwilling to tolerate awake surgery, including many children^5,13-17^.

Patient selection is essential. Wide-awake HSN requires reliable command following, tolerance of prolonged positioning, and reproducible intraoperative testing. Adjunct procedures should address residual dynamic imbalance consistent with the patient’s goals and consented operative scope. Local-anesthetic dose should be calculated before infiltration and monitored if the procedure is extended. No patient reached the dose ceiling, requested termination, or underwent conversion to GA, but these findings should not be generalized beyond appropriately selected patients.

Final between-cohort MAS differences ranged from 0.1 to 0.6 points. Most were below the contextual poststroke MCID of 0.48 to 0.76 points, while wrist-extensor and thumb-flexor differences reached its lower range^22^. Their clinical importance remains uncertain because MAS is ordinal, observations were nonindependent, and the cited MCID was derived from change in an averaged poststroke score rather than individual muscle groups after HSN.

### Limitations

This single-center, retrospective, nonrandomized study included 44 patients. Wide-awake eligibility depended on cooperation and procedural tolerance, while allocation also reflected age, cognition, anxiety, anesthetic suitability, and patient preference. These factors may affect rehabilitation and outcome assessment, creating selection bias and confounding by indication. Pooling adults and children further limit generalizability.

Most wide-awake procedures occurred later, and temporal or learning-curve effects could not be separated from the anesthetic strategy. The greater use of adjunct procedures in this cohort may also have influenced motion, posture, and strength. Multilevel procedures produced nonindependent observations, and the small subgroups precluded clustered or multivariable adjustment; joint-specific outcomes were therefore descriptive. Assessors were unblinded, and study-specific interobserver reliability was not evaluated.

The absence of major complications does not establish equivalent safety; the exact 95% CI upper limits were 16.1% after wide-awake surgery and 14.8% after GA. Secondary analyses lacked multiplicity adjustment, and operative-time and comparative patient-experience data were incomplete. Thus, these findings demonstrate feasibility and association, not anesthetic superiority. Prospective studies should use predefined eligibility, concurrent allocation, standardized adjunct-procedure criteria, blinded assessment, and methods accounting for multiple observations per patient.

## Conclusions

HSN under both anesthetic techniques was associated with sustained reduction in spasticity and preserved voluntary strength. Wide-awake HSN enabled real-time functional assessment and operative titration in selected cooperative patients, but its comparative benefit requires prospective verification.

## Funding

No funding.

## Declarations

The authors declare no competing interests.

## Author Contribution

J.T.J. Jerome, G. Surendran, K. Thirumagal: roles/writing—original draft, data curation, conceptualization, writing—drawing, review & editing.

## Appendix

Supporting material provided by the authors is posted with the online version of this article as a data supplement at jbjs.org (https://links.lww.com/JBJSOA/B383). This content was not copyedited or verified by JBJS.
